# Dietary Supplementation with Boswellia serrata, Verbascum thapsus, and Curcuma longa in Show Jumping Horses: Effects on Serum Proteome, Antioxidant Status, and Anti-Inflammatory Gene Expression

**DOI:** 10.3390/life13030750

**Published:** 2023-03-10

**Authors:** Daniela Beghelli, Lorenzo Zallocco, Cristina Angeloni, Onelia Bistoni, Maurizio Ronci, Clarita Cavallucci, Maria Rosa Mazzoni, Anna Nuccitelli, Chiara Catalano, Silvana Hrelia, Antonio Lucacchini, Laura Giusti

**Affiliations:** 1 School of Biosciences and Veterinary Medicine, University of Camerino, 62032 Camerino, Italy; 2 Department of Translational Research and New Technologies in Medicine and Surgery, University of Pisa, 56126 Pisa, Italy; 3 Department for Life Quality Studies, Alma Mater Studiorum, University of Bologna, 47921 Rimini, Italy; 4 Rheumatology Unit, Department of Medicine, University of Perugia, 06126 Perugia, Italy; 5 Department of Medical, Oral and Biotechnological Sciences, University of Chieti-Pescara, 66100 Chieti, Italy; 6 Independent Researcher, 06083 Perugia, Italy; 7 Department of Pharmacy, University of Pisa, 56126 Pisa, Italy; 8 Independent Researcher, 36023 Vicenza, Italy; 9 Department of Clinical and Experimental Medicine, University of Pisa, 56126 Pisa, Italy; 10 School of Pharmacy, University of Camerino, 62032 Camerino, Italy

**Keywords:** Boswellia serrata (Roxb ex Colebr), Verbascum thapsus, Curcuma longa, sport horses, serum proteome, oxidative stress, inflammation, immune responses

## Abstract

Intense exercise can cause inflammation and oxidative stress due to the production of reactive oxygen species. These pathophysiological processes are interdependent, and each one can induce the other, creating a vicious circle. A placebo-controlled blind study was carried out in show jumping horses (n. 16) to evaluate the effects of a commercial dietary supplement (Dolhorse^®^ N.B.F. Lanes srl, Milan, Italy) containing Verbascum thapsus leaf powder (1.42%), Curcuma longa (14.280 mg/kg), and Boswellia serrata (Roxb ex Colebr) (14.280 mg/kg) extracts. Before and after 10 days of dietary supplementation, blood samples were collected to evaluate the protein levels, antioxidants, and inflammatory responses by proteomic analysis or real-time Reverse Transcriptase-Polymerase Chain Reaction (real-time RT-PCR). A total of 36 protein spots, connected to 29 proteins, were modulated by dietary supplementation, whereas real-time RT-PCR revealed a significant downregulation of proinflammatory cytokines (interleukin 1α (*p* < 0.05) and interleukin-6 (0.005), toll-like receptor 4 (*p* < 0.05), and IKBKB (*p* < 0.05) in supplemented sport horses. Immunoglobulin chains, gelsolin, plasminogen, vitamin D binding protein, apolipoprotein AIV, and filamin B were overexpressed, whereas haptoglobin, α-2-HS-glycoprotein, α2-macroglobulin, afamin, amine oxidase, 60S acidic ribosomal protein, and complement fragments 3, 4, and 7 were reduced. No effect was observed on the antioxidant defense systems. The present results suggest this phytotherapy may reinforce the innate immune responses, thus representing a valid adjuvant to alleviate inflammation, which is a pathophysiological process in sport horses.

## 1. Introduction

Regular exercise induces physiological changes due to the high oxygen demands during training loads, which result in increased reactive oxygen species (ROS) formed from 1 to 2% of the oxygen that is not completely reduced into carbon dioxide and water [1,2,3].

Racehorses and competition horses experience high levels of physical exercise (both acute aerobic bouts and endurance) that are strictly connected to the occurrence of exercise-induced oxidative stress [4].

Indeed, the unbalanced equilibrium between oxidants and antioxidants due to the prevalence of the former results in oxidative stress that may have a negative influence on health, both in horses and athletes [5,6,7,8,9]. Furthermore, sport horses, during dressage, events, show jumping, endurance, and driving competitions, and athletes also experience inflammation because exercise elicits mechanical and hormonal reactions resulting in muscle damage. Therefore, inflammation arises as a physiological response to permit faster muscle regeneration after having removed the debris of damaged muscle cells [10,11]. The magnitude of the inflammatory process varies proportionately with exercise intensity and duration [11,12]. As the inflammatory process lasts for a longer time, the muscle damage can further propagate, resulting in increased injury and delayed recovery. This situation, in turn, aggravates oxidative stress and increases the production of ROS [13] which again play an important role in the progression of inflammatory disorders [14].

Nowadays, numerous studies have evidenced an interdependent relationship between inflammation and oxidative stress [15,16], whereas other studies have shown that oxidative stress could also play an important role as a therapeutic target as well as a prognostic index during serious pathologies, such as colitis, Equine Motor Neuron Disease, orthopedic pathologies, endometritis, and parasitic infestations [17,18,19].

Exercise load and diet are both factors that play a role in influencing the oxidative stress and antioxidant status of the equine athlete, and, thus, the effects of exogenous supplementation with different antioxidants have been studied extensively [3,20,21]. Supplementation of exogenous antioxidants such as vitamin E, selenium, methyl sulphonyl methane, alpha-lipoic acid, resveratrol, coenzyme Q10, and vitamin C has been tested as a possible tool to decrease the effects of exercise (endurance, jumping, and intense exercise) induced oxidative stress and increase the antioxidant status [22]. However, the results are rather controversial [21,23,24,25,26,27].

Regarding the use of plant extracts as feed additives with health benefits, peer-reviewed and scientific evidence of their usage in horses is lacking [28]. Indeed, even if trainers, breeders, horse owners, and veterinarians may use them as a traditional practice, reliable information on the usefulness and effects of herbal extracts in equines is scarce, as it is based on personal accounts rather than scientific evidence. An interesting article has been recently published on the use of milk thistle (Silybum marianum) in sport horses [29]. This plant, known for its anti-inflammatory, antioxidant, hepatoprotective, and neuroprotective properties, has revealed a possible positive effect on sport horse health and energy metabolism. However, milk thistle has not shown the ability to modulate antioxidant defenses, as evaluated by serum total antioxidant status and glutathione peroxidase activity.

The rhizome of Curcuma longa, which belongs to the Zingiberaceae family, contains a yellow pigment (curcumin), which represents the bioactive compound. Curcumin, however, has relatively low stability and low bioavailability due to its poor water solubility [30,31]. The small amount of curcumin absorbed systemically is mainly present in the conjugated form with glucuronic acid or sulfate instead of the free form. Many authors have described its antioxidant, anti-inflammatory, antibacterial, anti-cancer, and anti-atherosclerotic properties with this phytocomponent targeting the Nrf2 signaling pathway to protect cells against oxidative damage [32]. Experimental data have also shown that curcumin inhibits NF-κB activation in different tumor cell lines [33]. Some studies have reported the use of turmeric rhizome in horses for the treatment of hoof problems, arthritis, or placenta retention [34,35]. The combination of Curcuma longa and Boswellia serrata has been used for the treatment of arthritis in humans and dogs [36,37], even though some authors have questioned the positive health effects of curcumin due to its low bioavailability and failure to demonstrate a relationship between curcumin degradation products and metabolites and their potential health benefits [31]. Instead, other authors have just failed to highlight an overall anti-inflammatory effect in horses and ponies [38].

Modern medicine and pharmacology emphasize the use of the gum resin of Boswellia serrata, a branched tree of the Burseraceae family, as an anti-arthritic, anti-inflammatory, anti-hyperlipidemic, analgesic, and hepatoprotective compound. The resinous part of this plant grows in the mountainous and arid regions of India, North Africa, and the Middle East [39]. It contains monoterpenes, diterpenes, triterpenes, and triterpenic acids such as boswellic acids [40]. Clinical trials have confirmed that the anti-inflammatory activities of Boswellia serrata are attributable to the inhibition of iNOS, COX-2, NF-κB, and 5-lipoxygenase [41,42,43]. Some authors have tested Boswellia serrata in rugby athletes, where it is able to reduce inflammation [44]. However, in chronically exercising master athletes, Boswellia serrata, combined with curcumin, is unable to modulate inflammation, but only the glycol-oxidative status and lipid peroxidation [45].

Finally, Verbascum thapsus, known as common mullein, is a Eurasian plant belonging to the Scrophulariaceae family. It is widely used in folk medicine as a medicament for treating inflammatory diseases, such as asthma, allergies, arthritis, and arthrosis [46,47]. These plants are sources of numerous chemical compounds such as polysaccharides, iridoids, saponins, flavonoids, phenolic acids, and phenylethanoid glycosides, and among them the best known is the verbascoside or acteoside [48]. Verbascum thapsus has been used to counteract exercise-induced pulmonary hemorrhage in sport horses due to its expectorant and demulcent properties [49], but there are no studies proving its anti-inflammatory and antioxidant effectiveness in athletes and sport horses.

Although several studies have been carried out on the effects of antioxidant supplementation in horses during exercise, there is still a long way to go to completely understand the results obtained [3]. A better understanding and knowledge of the utilization of plant extracts in sports equines may dissipate the concerns over their usage, thus promoting them as feed additives to improve equine health and wellbeing [28]. Moreover, Boswellia serrata, Verbascum thapsus, and Curcuma longa have never been tested together for the beneficial effects on inflammatory and oxidative stress parameters of sport horses in controlled trials. The extracts of these plants may synergistically act to improve such parameters. 

The aim of this controlled study was to evaluate the antioxidant, anti-inflammatory, and immunomodulating activity of 10-day supplementation with a complementary feed containing Boswellia serrata (Roxb ex Colebr), Verbascum thapsus, and Curcuma longa (Dolhorse^®^) in sport horses (show jumping). 

Variations of serum protein levels were investigated using a proteomic approach, while the expression of inflammatory markers by peripheral blood mononuclear cells (PBMCs) was studied by real-time reverse transcriptase-polymerase chain reaction (real-time RT-PCR). In addition, total serum antioxidant capacity was also investigated.

## 2. Materials and Methods

### 2.1. Animals and Samples Collection

The study was approved by the Health Ministry (Directorate-General for Animal Health and Veterinary Medicines, Office 6), with protocol number: n° 238/2021-PR-, 30 March 2021. The placebo-controlled blind trial was carried out in a farm at Follonica (42°55′08″ N 10°45′41″ E), located in Tuscany, Italy, in December (mean temperature and humidity: 9 °C or 10 °C, and 59% or 83%, at T0 and T1, respectively). The study involved 16 show jumping horses of the Italian saddle breed that were randomly assigned to the dietary supplemented (DS) or placebo (control: CTRL) groups. Each group consisted of eight animals (4 geldings and 4 female mares each, mean ages of 10.63 ± 5.76 and 10.88 ± 6.24 years, respectively; body condition score: 3.0–3.2) [50]. Sport horses were housed in a well-ventilated stable within an individual pen (4 × 4 mt) and fed twice a day with the same diet consisting of hay ad libitum and concentrate feed, including cereal flakes and pellets, containing vitamins and minerals according to the Nutrient Requirements of Horses (National Research Council, 6th Edition, 2007) (more details in Supplementary Materials: Tables S1 and S2), and a water dispenser for ad libitum consumption. During the trial, they were regularly trained (moderate work for 40 min a day, 6 times a week, consisting of 20% walk, 50% trot, 15% canter, and 15% jump twice a week), but they did not participate in any official races. The experimental dietary supplement (Dolhorse^®^), which was kindly provided by N.b.f. Lanes srl (Milan, Italy), contained sunflower oil, 14.4% fish oil (35% EPA-25% DHA), 9.28% glucosamine, 7.13% chondroitin sulfate, 1.3% krill oil, synthetic vitamin E (35.69 gr/kg), in addition to Verbascum thapsus leaf powder (1.42%), Boswellia serrata (Roxb ex Colebr) and Curcuma longa extracts (14.28 g/kg each). The placebo, which was also prepared by N.B.F. Lanes srl, consisted of the same ingredients of Dolhorse^®^ without Verbascum thapsus, Boswellia serrata (Roxb ex Colebr) and Curcuma longa. During the 10-day trial, 70 mL of Dolhorse^®^ or placebo were administered daily with the morning feeding by the farm veterinarian. 

A total of 2 veterinarians collected blood samples by jugular venipuncture into a vacutainer (Vacutainer^®^, Becton Dickenson, Franklin Lakes, NJ, USA) with and without lithium heparin (10 mL/tube) at day 1 (T0) before starting the daily dietary supplementation and after 10 days (T1). The samples were shipped in cooling bags at 4 °C to the laboratory within 4 h and then immediately processed to separate serum and cells for further analysis.

### 2.2. Cells Stimulation and RNA Extraction

Peripheral blood mononuclear cells (PBMCs) were isolated from fresh heparinized blood samples by gradient centrifugation (800× *g* for 20 min). The number of live lymphocytes suspended in RPMI 1640 medium (Thermo Fisher Scientific, Waltham, MA, USA) with 10% heat-inactivated fetal bovine serum (Gibco TM, Thermo Fisher Scientific, Waltham, MA, USA), 100 units/mL penicillin (BiochromAG, Berlin, Germany), 100 μg/mL streptomycin (BiochromAG, Berlin, Germany), and 2 mM L-glutamine (Euroclone^®^, Pero Milan, Italy) complete medium was determined using a counting chamber and the trypan blue dye exclusion procedure [51]. The final concentration of live cells was adjusted to 4 × 10^6^/well (2 mL) and equal aliquots were treated with lipopolysaccharide (LPS: 100 ng/mL) (stimulated, s., cells) or vehicle (not stimulated, n.s., cells) for 2 h. Then, cells were washed twice with phosphate-buffered saline (GibcoTM, Thermo Fisher Scientific, Waltham, MA, USA) and finally stored at −80 °C until RNA extraction by Mini Kit (QIAGEN GmbH, Hilden, Germany). NanoVue SpeCTRLophotometer (GE Healthcare, Milan, Italy) was used to measure RNA yield and purity. Only samples with an A260/A280 ratio > 1.8 were used. 

### 2.3. Analysis of mRNA Levels by Real Time RT-PCR

For each sample, 1 μg of RNA was reverse transcribed to obtain cDNA using the iScript cDNA Synthesis Kit (Bio-Rad Laboratories, Hercules, CA, USA), following the manufacturer’s instructions. 

The subsequent PCR was performed in four sport horses/group in a total volume of 10 μL containing 2 μL of RNAsi-free dH_2_O, 2.5 μL (12.5 ng) of cDNA, 5 μL SsoAdvanced Universal SYBR Green Supermix (Bio-Rad Laboratories), and 0.5 μL (500 nM) of each primer. For each sample, three technical repeats were done. The investigated genes were interleukin-1α (IL-1α), -6 (IL-6), -10 (IL-10), toll-like receptor 4 (TLR4), interferon gamma (IFNγ), nuclear factor erythroid-2-related factor 2 (NFE2L2), superoxide dismutase (SOD), and I-kappa-B-kinase (IKBKB).

All primers were purchased from Sigma-Aldrich Life Science Co. LLC. (Burlington, MA, USA) and were intended for horse gene detection except for IKBKB, which was designed for the human gene and shows 94.3% homology with the horse ortholog. The list of primers is reported in Table 1. RN18S was used as a reference gene, and it was subsequently used to normalize Ct values. To evaluate the gene expression fold changes of s vs. ns lymphocytes of both DS and CTRL sport horses, the following formula was used:

(2^(−Δct)^) × 10,000

The formula was used as a relative quantification strategy for real-time RT-PCR data analysis [52].

### 2.4. Total Serum Antioxidant Capacity

Within 4 h after sampling, blood samples collected without anticoagulant were spun (2000× *g* for 10 min) to obtain serum that was stored at −80 °C until further analyses. Serum was used to evaluate the total antioxidant capacity by the 2,2′-azino-bis (3-ethylbenzothiazoline-6-sulfonic acid) (ABTS) (Sigma-Aldrich s.r.l.) and ferric reducing antioxidant power (FRAP) assays. The ABTS assay was essentially performed as reported in [53], but modified for application to a 96-well microplate [51]. Scalar dilutions of blood serum were mixed with the freshly prepared working solution of ABTS and incubated for 15 min at room temperature. Then the absorbance of each well was determined at 734 nm. 

The FRAP reagent (200 μL) was prepared as reported by Nkuimi Wandjou et al. [51] and mixed with a scalar dilution of blood serum (50 μL). After 15 min of reaction, the 96-well microplate was read at 593 nm.

In both assays, the total antioxidant capacity of blood serum was compared to Trolox (6-hydroxy-2,5,7,8-tetramethylchroman-2-carboxylic acid), used as a calibration standard, and expressed as mg of Trolox-equivalent antioxidant capacity for ml of serum (TEAC mg/mL) or μmol Trolox-equivalent for ml of serum (μmol TE/mL) in the ABTS and FRAP assays, respectively. Data are expressed as mean ± SD.

### 2.5. Proteomic Analysis

For proteomic analysis, sera were warmed up at 95 °C in 10% (*w/v*) SDS and 2.3% (*w/v*) DTT for 7 min, and after cooling were diluted in 2-D PAGE solubilization buffer [54]. Protein concentration was determined using the Pierce Protein Assay (Thermo Fisher Scientific, Waltham, MA, USA) with bovine serum albumin (BSA) as a standard. 

Two-dimensional gel electrophoresis (2-DE) was carried out as previously described [55]. Briefly, 150 μg of proteins were filled up to 100 μL in a rehydration solution. Isoelectrofocusing (IEF) was performed using 18-cm Serva IPG blue strips (SERVA-German Headquarter, Heidelberg, Germany) with a linear pH 3–10 gradient. IPG Blue Strips were rehydrated overnight in the absence of current in an Immobiline Dry Strip Reswelling Tray. IEF was carried out on the Ettan IPGphor Cup Loading Manifold, and after cup seating at the anodic end of the strip, samples were placed in the cups, and IEF immediately started (GE Healthcare, Uppsala, Sweden). The second dimension (SDS-PAGE) was carried out by transferring the proteins to 12% polyacrylamide gels. Gels were stained with 1 μM Ruthenium II tris (bathophenanthroline disulfonate) tetrasodium salt (RuBPS) (Cyanagen, Bologna, Italy) [56]. Images were acquired using ImageQuant LAS4010 (GE Health Care) and analyzed using Same Spot (V4.1, Total Lab, Newcastle Upon Tyne, UK) software as previously described [57]. Gel protein spots were excised and in-gel digested [58].

Trypsin-digested spots were analyzed by LC-MS/MS using an UltiMate3000 RSLCnano (Thermo Fisher Scientific, Waltham, MA, USA) chromatographic system coupled to an Orbitrap Fusion Tribrid mass spectrometer, operating in positive ionization mode and equipped with a nanoESI source (EASY-Spray NG) mass spectrometer (Thermo Fisher Scientific, Waltham, MA, USA). Peptides were loaded on a PepMap100 C18 pre-column cartridge (5 µm particle size, 100 Å pore size, 300 µm i.d. × 5 mm length, Thermo Fisher Scientific, Waltham, MA, USA) and then separated on an EASY-Spray PepMap RSLC C18 column (2 µm particle size, 100 Å pore size, 75 µm i.d. × 15 cm length, Thermo Fisher Scientific, Waltham, MA, USA) at a temperature of 38 °C and a flow rate of 300 nL/min, by a one-step linear gradient from 95% eluent A (0.1% FA in water) to 25% eluent B (99.9% ACN, 0.1% FA) in 56 min and a total LC run of 60 min. Precursor (MS1) survey scans were recorded in the Orbitrap at resolving powers of 120 K (at *m/z* 200). Data-dependent MS/MS (MS2) analysis was performed in top speed mode with a 3 s cycle time, during which the most abundant multiple-charged (2+–7+) precursor ions detected within the range of 375–1500 *m/z* were selected for activation in order of abundance and detected in the ion trap at a rapid scan rate. Quadrupole isolation with a 1.6 *m/z* isolation window was used, and dynamic exclusion was enabled for 60 s after a single scan. Automatic gain control targets were 4.0 × 105 for MS1 and 2.0 × 103 for MS2, with 50 and 300 ms maximum injection times, respectively. For MS2, the signal intensity threshold was 5.0 × 103, and the option “Injection Ions for All Available Parallelizable Time” was set. High-energy collisional dissociation (HCD) was performed using 30% normalized collision energy.

PEAKS Studio XPro software (Bioinformatic Solutions Inc., Waterloo, ON, Canada), using the “correct precursor only” option, was utilized to process raw data. The mass lists were searched against a custom protein database containing horse proteins (reviewed and unreviewed *Equus caballus* entries present in https://www.uniprot.org/, accessed on 5 October 2022 Searched Entry: 50616) and common MS contaminants, with no taxonomic restriction. Carbamidomethylation of cysteines was selected as a fixed modification and oxidation of methionines as a variable modification. Non-specific cleavage was allowed at one end of the peptides, with a maximum of 2 missed cleavages. Values of 10 ppm and 0.5 Da were set as the highest error mass tolerances for precursors and fragments, respectively. Peptide −10lgP was set at ≥35, corresponding to a peptide FDR lower than 0.01%.

### 2.6. Statistical Analysis

Data are presented as means ± SD. Student’s *t*-test or one-way ANOVA were used to compare differences among groups, followed by Bonferroni’s test (Prism 5, GraphPad Software, San Diego, CA, USA). *p* values < 0.05 were considered statistically significant. For proteomic experiments, statistical analysis was based on the normalized volume of each spot as measured by Same Spot software. A comparison analysis was performed between DS and CTRL images using one-way ANOVA. Spots that exhibited a fold change greater than 1.2 and a *p*-value < 0.05 and a *q*-value < 0.05 were taken into consideration for further protein identification. The number of horses (16), determined by power analysis (5% cut-off alpha: *p* < 0.05; beta 0.2; and power 0.8; Kane SP. Sample size calculator. ClinCalc: http:/calcclin.com/stats/samplesize.aspx accessed on 1 July 2017) ensured the correct number of samples for statistical analysis.

## 3. Results

Blood samples were analyzed and revealed that dietary supplementation was able to both modulate the expression of some serum proteins and downregulate some proinflammatory genes in lipopolysaccharide (LPS)-stimulated PBMCs.

### 3.1. Cellular Anti-Inflammatory and Antioxidant Activities

**Figure 1:**
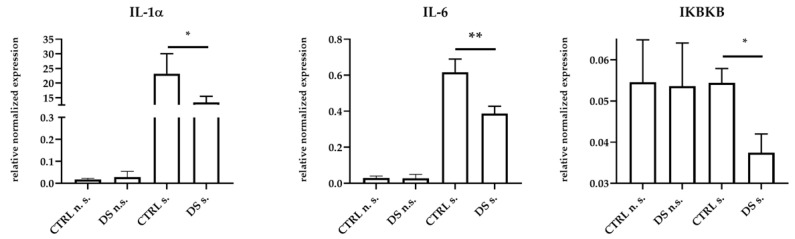
Real-time PCR quantification of mRNAs encoding IL-1α, IL-6, and IKBKB. PBMCs from 8 horses were treated with (s.) and without (n.s.) LPS for 2 h in an incubator at 37 °C, 5% CO_2._ Blood cells were collected at T0 and after 10 days (T1) of dietary supplementation with Dolhorse^®^ (4 DS horses) or placebo (4 CTRL horses). The T1 data are shown. Data are presented as means ± SD. Student’s *t*-test and one-way ANOVA followed by Bonferroni’s test were used to compare differences among groups. * *p <* 0.05, ** *p* < 0.005.

No statistically significant changes of IL-1α, IL-6, IFNγ, IL-10, IKBKB, and TLR4 expression were observed between the two groups of animals at T0 ), whereas significant changes were detected at T1. Thus, PBMCs from DS horses showed significant expression reductions of pro-inflammatory IL-1α, IL-6, and IKBKB genes compared to cells from CTRL horses (Figure 1). 

**Figure 2:**
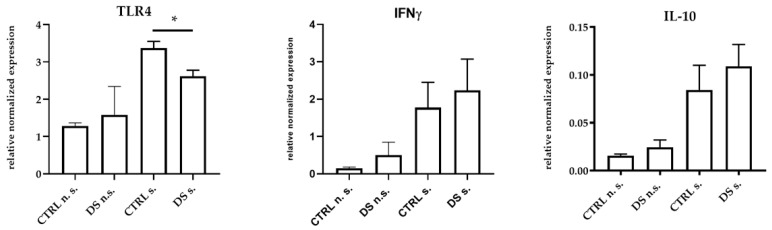
Real-time PCR quantification of mRNAs encoding TLR4, IFNγ, and IL-10. PBMCs from 8 horses were treated with (s.) and without (n.s.) LPS for 2 h in an incubator at 37 °C, 5% CO_2._ Blood cells were collected at T0 and after 10 days (T1) of dietary supplementation with Dolhorse^®^ (4 DS horses) or placebo (4 CTRL horses). The T1 data are shown. Data are presented as means ± SD. Student’s *t*-test and one-way ANOVA followed by Bonferroni’s test were used to compare differences among groups. * *p <* 0.05.

At the same time, DS was also able to significantly reduce TLR4 gene expression, while both IFNγ and IL-10 genes showed a trend toward higher expression, although not significant (Figure 2). Indeed, TLR4 is classically activated by LPS [59] through CD14, and upon its activation, TLR4 is able to trigger the production of pro-inflammatory mediators.

### 3.2. Cellular Antioxidant Activities

**Figure 3:**
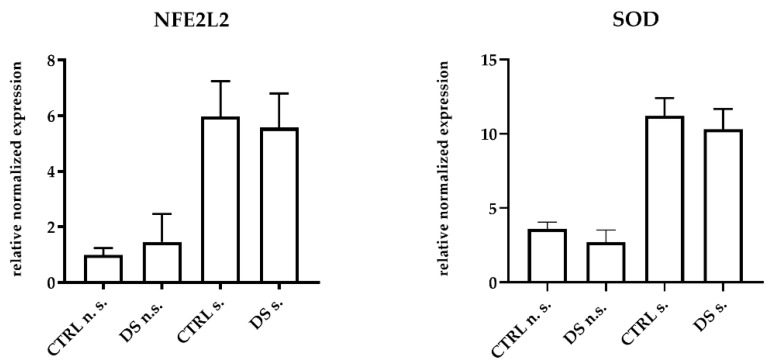
Real-time PCR quantification of mRNAs encoding NFE2L2, and SOD. PBMCs from 8 horses were treated with (s.) and without (n.s.) LPS for 2 h in an incubator at 37 °C, 5% CO_2._ Blood cells were collected at T0 and after 10 days (T1) of dietary supplementation with Dolhorse^®^ (4 DS horses) or placebo (4 CTRL horses). The T1 data are shown. Data are presented as means ± SD. Student’s *t*-test and one-way ANOVA followed by Bonferroni’s test were used to compare differences among groups.

DS was unable to modulate the expression of antioxidant genes and inducible enzymes in horse PBMCs. The mRNA expression levels of NFE2L2, the transcription factor responsible for antioxidant enzyme upregulation, and SOD, one of the main antioxidant enzymes, did not show any significant difference between groups at T0 ). At T1, DS and CTRL horses also expressed similar amounts of both transcripts in PBMCs, although the expression levels were clearly higher in LPS-stimulated cells (Figure 3). This result suggests that the 10-day treatment with DS did not affect the upstream regulator of oxidative stress.

### 3.3. In Vitro Antioxidant Capacity of Serum

**Figure 4:**
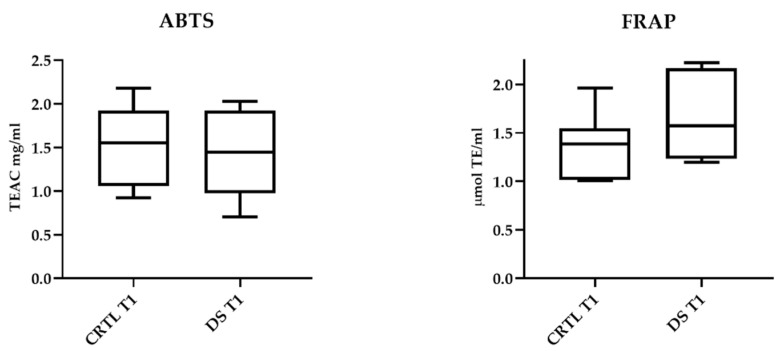
Serum antioxidant capacity of DS and CTRL horses after 10-day treatment. Blood samples were collected at T0 and after 10 days (T1) of dietary supplementation with Dolhorse^®^ (8 DS horses) or placebo (8 CTRL horses). In vitro ABTS and FRAP assays were used to measure serum antioxidant capacity. Data are expressed as mean ± SD. Student’s *t*-test was used to compare the differences between the horse groups.

ABTS and FRAP assays showed that 10-day DS does not affect serum antioxidant capacity. In Figure 4, the results relating to T1 samples of DS and CTRL horses are presented. No significant differences between the two horse groups were also detectable in T0 samples.

### 3.4. Serum Proteomic Analysis

**Figure 5:**
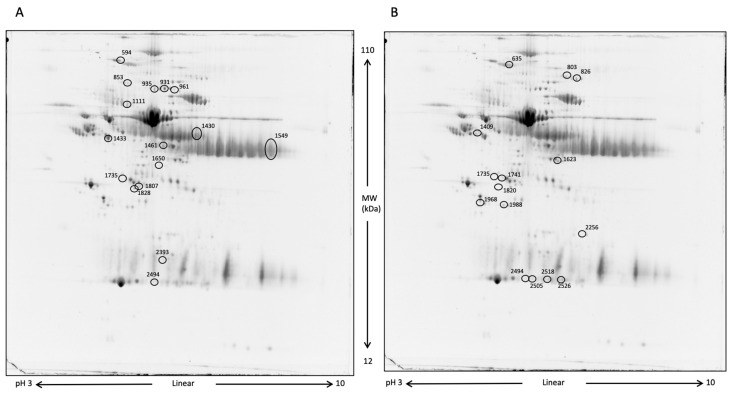
2-DE representative images of serum proteins. At first, proteins were separated by a 3–10 pH nonlinear gradient. Then, sodium dodecyl sulfate polyacrylamide gel electrophoresis (SDS-PAGE) was performed at 12% acrylamide. Gels were stained with Rutenium. (**A**), DS T1 (**left**) vs. DS T0 (**right**); (**B**), DS T1 (**right**) vs. CTRL T1 (**left**). Differentially expressed protein spots are circled.

Blood serum analysis aimed to determine whether differences in protein expression existed between the two groups of horses after 10 days of DS. The first comparative analysis was performed between DS and CTRL samples at T1, followed by a comparison of DS at T1 vs. DS at T0. Figure 5 shows representative two-dimensional electrophoresis (2-DE) separations of serum proteins. 

**Figure 6:**
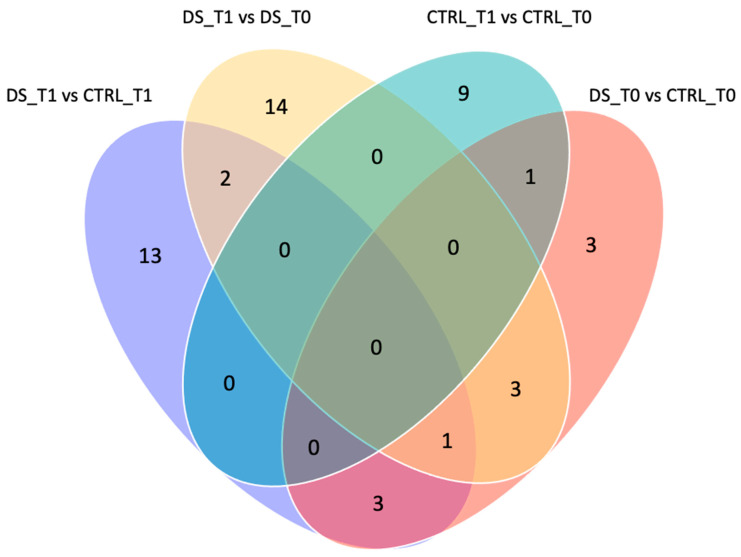
Venn diagram of different comparisons. Venn diagram highlighting the distribution of the identified differentially expressed spot proteins for each comparison. The overlapped and unique proteins are evidenced (Venny 2.0.2). Serum protein expression of 8 DS and 8 CTRL horses was compared between them at T0 (pink) and T1 (purple) and within each group before and after DS (light pink) or placebo (blue) treatment.

To exclusively evaluate the effect induced by DS and remove the variability inter- and intra-samples, only the spots that were significantly differentially expressed by the comparisons DS T1 vs. CTRL T1 and DS T1 vs. DS T0 were considered. The Venn diagram in Figure 6 shows the number of common and exclusive protein spots between the two comparisons. A total of 36 differentially expressed protein spots were detected at T1, of which 13 and 14 spots were detected in the comparison of DS T1 vs. CTRL T1 and DS T1 vs. DS T0, respectively.

Protein spots, which showed an expression fold change ≥ 1.2 were subsequently subjected to nano-LC-ESI-MS/MS analysis for their identification. The lists of differentially expressed proteins for T1 DS vs. T0 DS and T1 DS vs. T1 CTRL are shown in Table 2 and Table 3, respectively. In these tables, protein MW, pI, peptides, coverage values of MS/MS, ratios, and p-values are also presented. More than one identification was reported for some spots when MW and pI were not distinguishable.

The results obtained by proteomic analysis highlighted significant differences in the expression of some proteins after DS treatment. At T1, a general increase in the expression (ratio > 1.21–2.37) of Ig-like domain-containing proteins, vitamin D binding proteins, gelsolin, annexin, apolipoprotein A-IV, MHC class I antigens, and filamin B was observed in supplemented horses. In contrast, in the same horses, there was a general decrease in the expression (ratio < 0.48–0.9) of plasminogen, haptoglobin, α-2-HS-glycoprotein (also known as fetuin-A), α-2-macroglobulin, afamin, complement fractions 3 (C3), 4 (C4), and 7 (C7).

## 4. Discussion

The sport horse represents a very good in vivo model to study the effects of more or less intense physical activity, as it adapts perfectly to high physical efforts and is able to recover the optimal physical conditions in short periods [60].

Several studies have shown that there is an increase in inflammatory cytokines such as IL-1 and IL-6 in horse muscles and blood following exercise [61]. Furthermore, it should be emphasized that both exercise level and diet have a key role in modulating oxidative stress and antioxidant status in the equine athlete. In particular, it has been seen that physical activity leads to a reduction of oxidative stress markers and enhances the endogenous antioxidant defenses. 

In this study, the antioxidant, anti-inflammatory, and immunomodulating actions of a 10-day supplementation with a complementary feed containing or not *Boswellia serrata* (Roxb ex Colebr), *Verbascum thapsus*, and *Curcuma longa* (Dolhorse^®^) in sport horses (show jumping) were evaluated.

Surprisingly, no increase of cell and serum antioxidant defenses were observed in dietary supplemented horses, as suggested by the absence of changes in NFE2L2 and SOD mRNA levels and the expression of antioxidant proteins. This lack of antioxidant defense responses could originate from a true absence of an oxidative stress condition in both animal groups. We cannot exclude this possibility in light of the fact that Ashrafizadeh et al. have recently reported that curcumin induces activation of the NFE2L2/Rnf2 pathway [32]. 

On the contrary, the diet affected a complex set of signaling pathways that interplayed with the immune responses. Indeed, a significant decrease of IKBKB and TLR4 expression was observed concomitantly with a decrease in some pro-inflammatory cytokines such as IL-1α and IL-6 transcripts. Toll-like receptor 4 represents one of the TLRs, such as TLR3 and TLR9, which recognize LPS stimulus to initiate the innate and adaptive immune responses. The trigger of TLRs happens through the recruitment of different adaptor family members, such as primarily myeloid differentiation primary response 88 (MyD88) and TIR domain-containing adaptor protein-inducing IFNβ (TRIF). The first signaling pathway mediated by MyD88 culminates in NF-κB and MAP kinase activation [62], resulting, in the first instance, in the induction of inflammatory genes such as IL-6, TNFα, and IL-1β, and, only relatively later, in the induction of IL-10 synthesis. Toll-like receptor 4 uniquely utilizes both MyD88 and TRIF to regulate the production of pro-inflammatory cytokines and type I interferons (I IFNs) [63]. IFN signaling represents a requirement for the induction of anti-inflammatory cytokines such as IL-10 and IL-27 [64]. In fact, LPS-induced type I IFN in macrophages contributes to IL-10 mRNA expression and stabilization, whose prolonged action serves to control the adverse effects of inflammation on the host by regulating proinflammatory cytokine production [65].

In the present study, the reduced expression of TLR4 detected in DS horses likely causes a decrease in signaling through the MyD88 pathway, which may, in turn, lead to reduced IkBKB expression, a consequent decrease in NF-kB activation, and in the end, lower expression of pro-inflammatory cytokines IL-1α and IL-6. On the contrary, the TRIF pathway does not seem to be affected by DS since LPS-induced expression of IL-10, a classical anti-inflammatory cytokine [62], and IFNγ mRNA are similar in DS-treated and CTRL horses. Studies in vivo on various animal models have highlighted that curcumin and even boswellic acids are able to reduce the expression of TLR4, MyD88, and NF-kB [66,67].

In DS horses, the serum proteomic analysis evidenced significant expression differences of various proteins, of which many are involved in immune responses. The light chain λ of the immunoglobulins appeared slightly less and more expressed by comparing T1 DS vs. T0DS and T1 DS vs. T1CTRL, respectively. The heavy chain µ, which is the characteristic IgM heavy chain, also resulted in downregulation in horses after 10 days of DS treatment (T1 DS vs. T0DS). These results suggest a decrease in blood IgM antibodies of the primary response, which are excellent activators of the classical complement pathway leading to the lysis of opsonized bacteria or foreign cells. A concomitant reduction of three complement components, C3, C4, and C7, further supports the modulatory effect of the DS-treatment on the immune response. Whereas C4 and C7 expressions were decreased in T1 DS compared to T1CTRL, C3 was reduced in T1 DS compared to both T0DS and T1CTRL. Processing of C3 by C3-convertase is the central reaction of classical and alternative complement activation pathways, key systems of innate and adaptive immune responses. In rheumatoid arthritis patients treated for 12 months with tocilizumab, an anti-interleukin-6 receptor monoclonal antibody that rapidly reduces acute phase reactants, C3 and C4 serum levels also decrease [68]. Based on this report, we might suppose a link between DS-induced IL-6 reduced expression and decreased C3 and C4 serum levels in supplemented horses.

Modulation of immune responses by DS is also highlighted by the increased levels of Ig-like domain-containing proteins and major histocompatibility complex class I protein (MHC class I antigen) in T1 DS horses compared to T0DS and T1CTRL. Many plasma membrane proteins of leukocytes are built up from immunoglobulin-like domains, including T (TcR) and B cell antigen receptors [69]. The MHC class I antigen, expressed on the plasma membrane of all nucleated cells, is made up of a transmembrane chain and non-covalently linked β2 microglobulin consisting of three extracellular domains and one domain, respectively. The proximal domain of the heavy chain and β2 microglobulin have an Ig-like domain structure. Major histocompatibility complex class I antigen protein, B cell antigen receptor, and TcR have different specialized functions, which play pivotal roles in the adaptive immune response. It could be speculated that the increased serum levels of Ig-like domain-containing proteins and MHC class I antigen are the outcome of their enhanced shedding in T1 DS horses. Indeed, β2-free MHC class I molecules have been reported to be selectively cleaved and released from the plasma membrane by membrane metalloproteinases [70].

The observed increase of serum vitamin D binding protein (DBP) in horses at T1 DS vs. T0DS is also noteworthy for the emerging role of the protein in the innate immune response. This glycoprotein, a member of the albuminoid family, is involved in the transport of vitamin D and its metabolites, as well as in the scavenging of extracellular G-actin. DBP has no direct effects on inflammation, although it indirectly influences both inflammation and immune responses by preventing ligand entry into cells. A vitamin D-binding protein also plays a more direct role in the inflammatory cascade by binding to activated leukocyte membranes and in association with annexin 2 (ANXA2), enhancing complement C5a-stimulated chemotactic activity [71].

Higher serum levels of ANXA2, filamin B (FLNB), and gelsolin (GSN) were also observed in horses after DS compared to T0. ANXA2, a secreted and extracellular matrix protein produced by a wide array of cell types, including monocytes and macrophages, is a well-established hemostasis regulator. This protein does not only contribute to fibrinolysis but also promotes proteolysis of the extracellular matrix, regulates inflammation and immune system activation, and facilitates tissue injury and repair [72]. Even though ANXA2 is considered a pro-inflammatory factor in autoimmune diseases such as rheumatoid arthritis [72], in supplemented horses, its increased expression could facilitate the migration of immunocompetent cells through tissues. This action, in concert with those operated by FLNB, GSN, and DBP, could promote a greater efficiency of the immune surveillance process operated by both innate and adaptive immune systems. FLNB is a cytoplasmic and cytoskeletal protein that plays a crucial role as a regulator of intercellular adhesion molecule 1 (ICAM-1) function in the process of transendothelial migration of leukocytes. Since FLNs are the most potent F-actin crosslinkers [73], it is not surprising that FLNB is pivotal in immune surveillance and inflammation [74]. Likewise, GSN is involved in chemotaxis, tissue remodeling, and phagocytosis, which are processes occurring during tissue inflammation [75]. Based on its considerable expression decrease in pathologies characterized by a chronic inflammatory state such as arthritis rheumatoid, it has been postulated that GSN plays a protective role in inflammation [76]. 

Apolipoprotein A-IV was also noticed to be overexpressed in the serum of horses that received the supplementation by comparing T1 DS vs. T0 DS. Apolipoproteins A-IV are important components of the lipoprotein particles that transport cholesterol, triglycerides, and phospholipids through the blood from and toward different tissues. Indeed, lipoproteins have the ability to suppress inflammation, oxidative stress, tissue remodeling, and promote adaptive immunity and host defense [77,78,79]. Other proteins were observed at lower levels in the serum of T1 DS horses compared to basal or T1CTRL horses. Whereas afamin (AFM), amino oxidase (AOC3), and actin γ1 (ACTG1) resulted in decreased levels in the T1 DS vs. T0DS comparison, α-2-macroglobulin (A2M), plasminogen (PLG), α-2-HS-glycoprotein (AHSG), IF rod domain-containing protein (KRT18), haptoglobin, and 60S acidic ribosomal protein P0 (RPLP0) were discovered to be reduced in the T1 DS vs. T1CTRL comparison. 

A member of the albuminoid family, AFM, is a serum 75 kDa-glycoprotein that is also present in other body fluids and exhibits vitamin E binding properties [80]. In humans, elevated serum AFM levels may suggest a state of oxidative stress and inflammation strongly associated with insulin resistance in polycystic ovary syndrome [81], while increased levels have also been detected by mass spectrometry in the uterine fluid of early pregnant horses with insulin resistance compared to animals without insulin resistance. Moreover, dietary omega-3 supplementation, which is endowed with an anti-inflammatory activity, has been shown to reduce AFM levels [82]. This may suggest that our dietary supplementation has a similar effect. 

The amine oxidase, copper-containing 3 (AOC3), also called semicarbazide-sensitive amine oxidase (SSAO), plasma amine oxidase (PAO), vascular adhesion protein-1 (VAP-1), and primary amine oxidase, represents the most studied of the three human CAOs [83]. A type II transmembrane glycoprotein, AOC3, is mainly observed in adipocytes, smooth muscle, and endothelial cells. A soluble form is released upon cleavage of the C-terminus by a metalloprotease. In healthy humans, serum levels of soluble AOC3 are low, while increased levels have been observed in the sera of patients suffering from diabetes, heart failure, and liver diseases. Both an adhesion domain and the amine oxidase activity of AOC3 are critical for AOC3-mediated induction of leukocyte rolling, adhesion, and transmigration in response to inflammatory stimuli [84]. Studies both in vitro and in vivo have highlighted that AOC3 inhibition can be an effective therapeutic approach to treat inflammatory and fibrotic diseases [83]. Thus, the DS-induced reduction of AOC3 serum levels corroborates the anti-inflammatory activity of our supplementation once again. 

The plasma glycoprotein A2M is a protease inhibitor with a high affinity and broad specificity. A potential role of A2M in inflammation, immunity, and infections has been recognized [85]. In inflammation A2M clearly protects against structural damage inhibiting proteases released by activated leukocytes but some evidence also indicates that its function is far more complex than just protease inhibition. In fact, this glycoprotein can interact with many factors involved in inflammation such as the proteins of the complement system, growth factors, cytokines, chemokines, and cell-surface receptors thus modulating leukocytes functions [85]. In human, A2M, haptoglobin, and complement factors are included among the positive acute phase proteins since their serum levels rise during acute inflammation. Hence, A2M decreased levels in DS horses is another evidence supporting the anti-inflammatory effect of the dietary phytotherapy. 

Finally, PLG and AHSG (fetuin-A), which are reduced in DS-treated horses, are plasma glycoproteins, which too play a role in inflammation. Whereas PLG is a zymogen whose activation leads to the generation of plasmin, a broad-spectrum serine protease, fetuin-A functions as a carrier and multifunctional protein that participates in a wide array of essential biological activities such as regulation of bone and calcium metabolism and the insulin signaling pathway [86,87]. In the inflammatory response, PLG and plasmin carry out numerous functions such as intravascular and extravascular fibrinolysis, stimulation of leukocyte migration, extracellular matrix degradation, and participation in the wound healing process. Plasminogen also interacts with several complement components, including C3, C3b, C5, and C4bP, and while plasmin degrades both C3b and C5, it acts as an inhibitory regulator of the complement cascade [86,88,89]. Concerning the role of fetuin-A in the inflammatory process, increasing evidence points out that this glycoprotein has both pro-inflammatory and anti-inflammatory activity depending on the stimulus that triggers its production. Indeed, fetuin-A is considered a negative acute-phase protein that quickly decreases in response to acute inflammation [87]. On the other hand, numerous clinical studies have also revealed the pro-inflammatory effects of this protein by finding increased levels in patients suffering from diseases in which an inflammatory condition exists, such as obesity, metabolic syndrome, insulin resistance, type 2 diabetes (T2D), atherosclerosis, and non-alcoholic fatty liver disease (NAFLD). Since it has been reported that dietary supplementation of curcumin significantly reduces fetuin-A serum levels in rats fed with a high-fat diet, it could be speculated that the presence of this photoelement in our DS concurs in lowering fetuin-A levels in horses, too [90]. 

## 5. Conclusions

Overall, the findings of the present study demonstrate an action of dietary supplementation in containing LPS-induced inflammatory responses in leukocytes and modulating the expression of various blood proteins involved in inflammation and immune responses. Therefore, the proposed dietary supplementation may be considered a useful phytotherapy to reduce inflammation and innate immunity activation triggered by intense exercise in sports horses. The study has also contributed to improving our understanding of the molecules and molecular mechanisms implicated in the activity of such supplementation. 

In further studies, it would be interesting to assess, immediately after exercise, whether this supplement can modify the antioxidant and immune responses investigated here and whether different metabolic pathways are involved in relation to sport discipline types. The anti-inflammatory and immunomodulating activities of every single plant also need to be investigated to understand both potency and synergistic effects. 

Moreover, at present, it is not known how long the effects of this dietary supplementation will persist or whether they are only temporary effects.

Monitoring the duration of these effects over time and/or determining whether the increase in the duration of administration can provide superior results will certainly be the object of further studies.

## Figures and Tables

**Table 1:** List of primers (Sigma-Aldrich Life Science Co. LLC., USA) utilized in the present study.

Gene	5′-Forward-3′	5′-Reverse-3′
IL-1α	TTGAGTCGGCAAAGAAATC	GAGAGAGATGGTCAATTTCAG
IL-6	CAGCACATTAAGTACATCCTC	AAAGACCAGTGGTGATTTTC
IL-10	CAGGGTGAAGACTTTCTTTC	AAACTGGATCATCTCCGAC
TLR4	CAGAAAATGCCAGGATGATG	TAGAGATTCAGGTCCATGC
NFE2L2	CAACACATCTCATCAGAACC	GGAGAAACCTCATTGTCATC
IFNγ	AGAACTGGGAAAGAGGATAGTG	ATGGCTCTTTTGAATGACCTG
IKBKB	ATGAATGCCTCTCGACTTAG	CCAGTTCTTCACTCTTCTTG
SOD	CCTATGTGAACAACCTGAAC	CTCCACCATTGAACTTGAG
RN18S	CAATACAGGACTCTTTCGAG	ATATACGCTATTGGAGCTGG

**Table 2:** List of exclusive proteins differentially expressed in T1 DS horses compared to T0 DS.

Spot n.	ID	Gene	Description	Cov. (%)	#Pept.	#Uniq.	MW	pI	Ratio DS_T1/DS_T0
594	A0A3Q2H1S8	FLNB	Filamin B	1	1	1	275,363	5.5	1.33
594	F6XT77	FLNB	Filamin B	1	1	1	277,858	5.46	
594	A0A5F5PVA4	FLNB	Filamin B	1	1	1	281,354	5.42	
853	F7DLT3	AOC3	Amine oxidase	1	1	1	84,500	5.93	0.70
853	A0A5F5PLA5	AOC3	Amine oxidase	1	1	1	84,561	5.98	
853	F6T7X3	AOC3	Amine oxidase	1	1	1	84,745	5.93	
931	A0A3Q2H4N7	GSN	Gelsolin	13	10	10	82,588	5.34	1.22
935	Q28372	GSN	Gelsolin	5	5	5	80,827	5.58	1.25
961	F7E2D1	GSN	Gelsolin	6	5	5	580,745	5.64	1.21
1111	F7B3I5	AFM	Afamin	3	2	2	68,871	5.49	0.73
1111	A0A3Q2ID60	AFM	Afamin	3	2	2	71,279	5.48	
1430	H9GZQ9	-	Immunoglobulin heavy constant µ	6	4	2	48,112	6.64	0.76
1433	F6T0P6	GC	Vitamin D binding protein	15	7	7	54,327	5.46	1.35
1461	H9GZT5	-	Immunoglobulin heavy constant µ	2	1	1	60,138	6.17	1.23
1549	H9GZU8	-	Immunoglobulin heavy constant µ	9	3	2	48,837	7.5	0.80
1650	A0A3Q2LPE6	ANXA2	Annexin	14	6	6	44,464	6.68	1.24
1735	A0A3Q2HWQ6	LOC100060505	Complement C3	1	2	2	186,448		0.69
1807	F7AAK7	ACTG1	Actin γ 1	15	4	4	41,793	5.31	0.80
1828	F6RZ27	APOA4	Apolipoprotein A-IV	25	10	10	43,252	5.38	1.49
2494	A0A3Q2HMJ3	-	Ig-like domain-containing protein	20	3	1	14,975	8.58	1.89
2393	A0A0A1E6N9	IGL	Immunoglobulin λ light chain variable reg	11	2	1	22,794	5.11	0.89

Spot n.—number of the spot; ID—uniport identification number; Gene—gene name; Description—protein name; Cov. (%)—percent of coverage; # Pept.—number of peptides; # Uniq.—number of unique peptides; MW—molecular weight; pI—isoelectric point.

**Table 3:** List of exclusive proteins differentially expressed in DS horses at T1 compared to CTRL horses at T1.

Spot n.	ID	Gene	Protein Name	Cov. (%)	#Pept.	#Uniq.	MW	pI	Ratio DS_T1/CTRL_T1
635	F6RI47	A2M	α-2-macroglobulin	1	1	1	161,000	6.17	0.73
803	A0A3Q2L7R0	PLG	Plasminogen	4	5	5	91,934	6.72	0.48
826	A0A3Q2KNA7	C7	Complement C7	2	2	2	93,001	6.61	0.51
1409	F7C450	AHSG	α-2-HS glycoprotein	6	2	2	38,724	5.68	0.59
1623	A0A3Q2I440	KRT18	IF rod domain-containing protein	4	2	1	48,300	5.74	0.83
1735	A0A3Q2HWQ6	LOC100060505	Complement C3	1	2	2	186,448		0.69
1741	A0A3Q2HWQ6	LOC100060505	Complement C3	4	8	8	186,448		0.73
1820	F6XWM5	LOC100067869	Haptoglobin	3	1	1	38,466	5.59	0.56
1968	D9MNN2	Eqca-N	MHC class I antigen (Fragment)	6	2	2	34,847	5.28	2.37
1988	F6TTP1	RPLP0	60S acidic ribomal protein P0	3	1	1	34,288	5.7	0.88
2256	F6XSF7	LOC100059239	Complement C4 γ chain	3	5	5	174,058	6.17	0.61
2494	A0A3Q2HMJ3	-	Ig-like domain-containing protein	20	3	1	14,975	8.58	1.89
2505	A0A0A1E6K7	IGL	Immunoglobulin λ light chain variable reg	11	2	1	23,195		2.00
2518	A0A5F5PPG4	-	Ig-like domain-containing protein	6	1	1	17,340	6.99	1.73
2526	A0A3Q2H7F5	-	Ig-like domain-containing protein	6	1	1	16,902		1.36

Spot n.—number of the spot; ID—uniport identification number; Gene—gene name; Description—protein name; Cov. (%)—percent of coverage; # Pept.—number of peptides; # Uniq.—number of unique peptides; MW—molecular weight; pI—isoelectric point.

## Data Availability

Data supporting the results of this study are available from authors and are available on request.

## Supplementary Materials

The following supporting information can be downloaded at: https://www.mdpi.com/article/10.3390/life13030750/s1, Table S1. Composition of concentrate, Table S2. Additives for kg of concentrate.
