# Supplementary material for: Dietary Supplementation with Boswellia serrata, Verbascum thapsus, and Curcuma longa in Show Jumping Horses: Effects on Serum Proteome, Antioxidant Status, and Anti-Inflammatory Gene Expression
Source: Life (Basel). 2023 Mar 10;13(3):750. doi: 10.3390/life13030750 (PMC10055707; doi:10.3390/life13030750)
Supplement: Supplementary file 1 [file life-13-00750-s001.zip › life-2231138-supplementary.pdf]

Concentrate	%
Moisture	12.50%
DE kcal	2890
Crude protein	14.00%
Crude fat	3.00 %
Crude fiber	14.00%
Ash	9.00%
Calcium	1.20%
Phosphorus	0.60%

Table S1. Composition of concentrate.

Additives		
Vitamin A	30000	U.I.
Vitamin D_3	2000	U.I.
Vitamin E	30	mg
Vitamin K	0.8	mg
Vitamin B_1	2	mg
Vitamin B_2	1	mg
Vitamin B_{12}	0.01	mg
Vitamin PP	130	mg
Pantotenic Acid	0.8	mg
Iron	50	mg
Manganese	50	mg
Copper	30	mg
Zinc	80	mg
Iodine	2.0	mg
Organic Selenium	0.1	mg
Lysine	7000	mg
Methionine	2500	mg

Table S2. Additives for kg of concentrate.

The feeding ration was comprised of grass hay (first-cut), administered two or three times a day, and 3-3.5 kg of concentrate administered two times/day. The feeding ration might have been slightly varied according to the animal's requirement.
